# Single breathhold three-dimensional cardiac cine MRI with whole ventricular coverage and retrospective cardiac gating using kat ARC

**DOI:** 10.1186/1532-429X-14-S1-W69

**Published:** 2012-02-01

**Authors:** Peng Lai, Maggie M Fung, Shreyas S Vasanawala, Anja C Brau

**Affiliations:** 1 Global Applied Science Laboratory, GE Healthcare, Menlo Park, CA, USA; 2 MR Clinical Development Team, GE Healthcare, Waukesha, WI, USA; 3 Radiology, Stanford University, Stanford, CA, USA

## Summary

This work presents a kt acceleration method (kat ARC) for retrospective cardiac-gated 3D cardiac cine MRI. Our in-vivo results show that 3D cine images of the entire ventricle are obtainable within a single breathhold using highly accelerated kat ARC.

## Background

For quantitative volumetric assessments of cardiac function, 3D cine images depicting motion of the entire ventricle in a complete cardiac cycle are needed. However, due to limited acceleration capability, breathheld 3D cine MRI is not obtainable using conventional parallel imaging. Several kt-acceleration methods have demonstrated high-acceleration capability for dynamic MRI by exploiting spatiotemporal correlation [Tsao, MRM 2003; Huang, MRM 2005]. This study aims to investigate the feasibility of breathheld retrospective cardiac-gated whole-ventricle 3D cine MRI using kat ARC (k-adaptive-t Autocalibrating Reconstruction for Cartesian sampling [Lai, ISMRM 2009]).

## Methods

**Figure 1:**
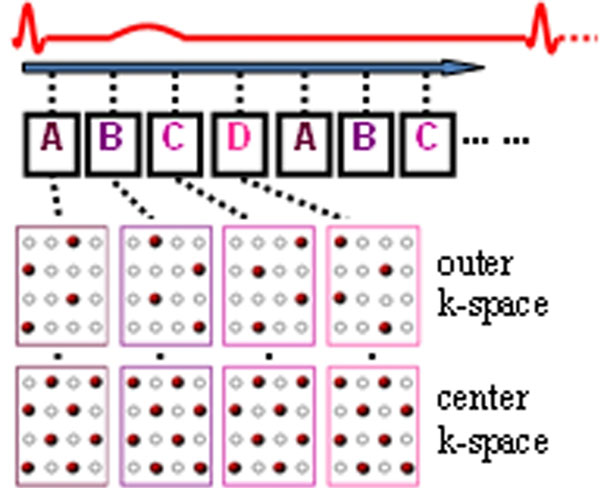
kat ARC acquisition with 4× outer and 2× center acceleration. A, B, C, D indicate different sampling patterns.

Fig 1 demonstrates the acquisition scheme of the proposed kat ARC method. Time-interleaved undersampling was performed in both outer and center k-space with different acceleration. For accurate reconstruction, low acceleration was used in center k-space. For fast imaging, high acceleration was used in outer k-space. The sampling pattern in both k-space regions was optimized by maximizing spatiotemporal correlation between sampled and unsampled data. The image was reconstructed using the following steps: 1. Retrospective cardiac gating of acquired k-space data based on simultaneously recorded cardiac signals; 2. Completing center k-space lines using tGRAPPA [Breuer, MRM 2005]; 3. Synthesizing unsampled outer k-space lines using kat ARC [3]. To preserve temporal fidelity in kat ARC reconstruction, local cardiac motion around each cardiac phase was estimated on low resolution images from center k-space data and a phase-specific temporal window was determined for each cardiac phase, selecting the most consistent temporal neighbors for data synthesis.

Healthy volunteers were scanned on a GE 1.5T scanner with a 32-channel cardiac coil. 3D cine images were collected from a short-axis slab covering the entire ventricle (2.5×2.5mm^2^ in-plane resolution, 24 slices with 5mm thickness, 48ms temporal resolution). Each volunteer was scanned 3 times using different acceleration: A. Reference with 6× (~30 sec): 3×2 outer and 2×1 center acceleration; B. 8× (~20 sec): 4×2 outer and 3×1 center acceleration; C. 10× (~16 sec): 5×2 outer and 2×2 center acceleration. High acceleration images were compared with the reference image with regard to overall image quality and motion depiction.

## Results

**Figure 2:**
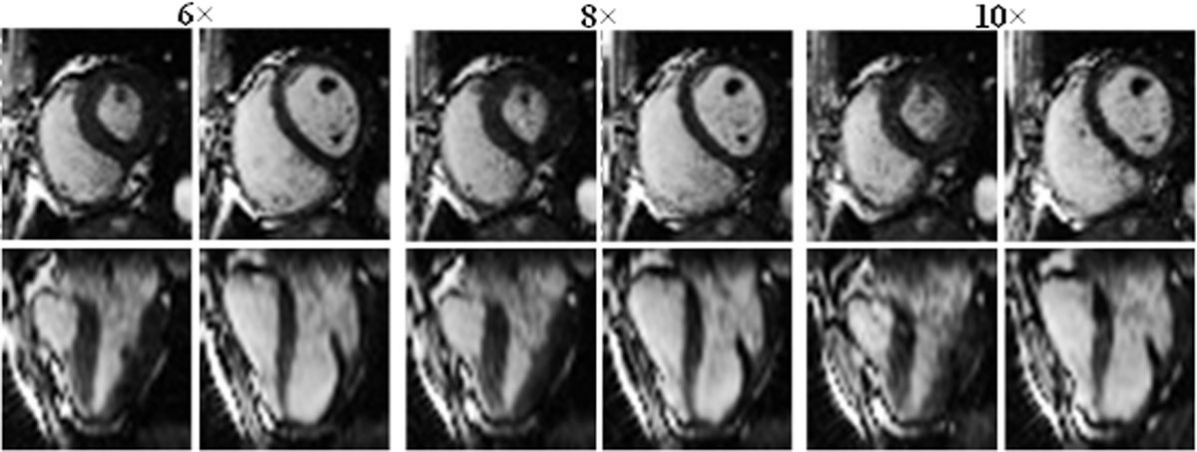
3D cardiac images in the original plane (upper) and a reformatted plane (lower) acquired with 6×, 8× & 10× at mid-systole (left) and mid-diastole (right). Note: breathhold position is slightly shifted in different acquisitions.

Fig 2 shows a representative example comparing images collected from the same subject. The 8× image is very similar to the reference image with 6× at both mid-systole & diastole. Though 10× generates mild artifacts, its delineation of myocardial edge is not significantly compromised, enabling accurate motion and volume assessments.

## Conclusions

Based on our in vivo results, single breathhold imaging of whole-ventricular cardiac motion is achievable using highly accelerated kat ARC. Future work will further optimize imaging parameters and sampling pattern to improve image quality at high acceleration and conduct clinical evaluations.

